# Less is more: comparison of automated spiral-drawing metrics with clinician-rated tremor scores after MRgFUS thalamotomy

**DOI:** 10.3389/fneur.2026.1848802

**Published:** 2026-07-29

**Authors:** Mark Katson, Teddy Lazebnik, Meirav Har-Even, Maxim Glebov, Fawzia Naamnih, Alon Sinai, Maria Nassar, Inna Senderova, Marius Constantinescu, Lior Lev Tov, Vered Aharonson, Ilana Schlesinger

**Affiliations:** 1 Department of Neurology, Rambam Health Care Campus, Haifa, Israel; 2 Department of Information Systems, University of Haifa, Haifa, Israel; 3 Department of Computing, Jonkoping University, Jonkoping, Sweden; 4 Department of Anesthesiology, Sheba Medical Center, Ramat Gan, Israel; 5 Department of Neurosurgery, Rambam Health Care Campus, Haifa, Israel; 6 School of Electrical and Information Engineering, University of the Witwatersrand, Johannesburg, South Africa; 7 Medical School, University of Nicosia, Nicosia, Cyprus; 8 Rappaport Faculty of Medicine, Technion-Israel Institute of Technology, Haifa, Israel

**Keywords:** clinical computer vision, essential tremor, image-based biomarkers, model evaluation and validation, MRI guided focused ultrasound, spiral drawing analysis

## Abstract

Objective measures of tremor could improve assessment of response to magnetic resonance-guided focused ultrasound (MRgFUS) for essential tremor in practice, but it is unclear whether image-derived metrics from spiral drawings reflect clinician judgment. In this study, we analyzed 410 spiral-drawing observations from 106 patients with essential tremor undergoing MRgFUS thalamotomy and compared two recent computer-vision measures with a simple geometric metric (total line length) against an ordinal clinical rating scale for tremor spiral scores using ordered logistic mixed-effects models. Only line length independently tracked worse clinician-rated severity (odds ratio_(per 1 mm)_ 1.017, *p* < 0.01), whereas the two image-based scores showed no significant association. The model captured the expected treatment signature, with marked post-procedural improvement confined to the treated hand. These findings suggest that low-complexity interpretable features may better approximate clinically meaningful tremor severity than more elaborate image-processing metrics and support further validation against functional outcomes.

## 1 Introduction

Essential tremor (ET) is the most common movement disorder, typically manifesting as postural and kinetic tremor, most commonly of the upper extremities, that can substantially impair activities of daily living, including eating, drinking, and handwriting (1). ET affects roughly 0.9% of the population overall and increases with age (reported at a rate of 4.6% in individuals over 65 years of age). As the condition progresses, many patients show insufficient response to pharmacologic therapies (2). For these refractory patients, lesioning or neuromodulation has long been an effective strategy.

Magnetic resonance-guided focused ultrasound (MRgFUS) thalamotomy has emerged as a minimally invasive alternative to more invasive functional neurosurgical approaches for tremor (3–5). MRgFUS delivers acoustic energy through the skull to thermally ablate tissue, while MRI provides stereotactic planning and real-time thermometry (6). In ET, the procedure commonly targets the ventral intermediate nucleus (VIM), a key cerebellar motor relay component implicated in tremor generation. During treatment, sonications are typically escalated stepwise with repeated bedside examinations to verify tremor improvement and monitor adverse effects (7). This highlights a practical need: objective, sensitive tremor quantification that can complement clinical examination before and after intervention, and if possible, also during intervention.

In routine care and trials, tremor severity is commonly captured using clinician-rated scales such as the Clinical Rating Scale for Tremor (CRST; also referred to as the Fahn–Tolosa–Marin tremor rating scale), which incorporates drawing tasks (e.g., Archimedes spirals and lines) (8, 9). The CRST spiral score uses a 0–4 ordinal scale that captures the clinician's global visual assessment of tremor severity during spiral drawing, integrating features such as oscillation amplitude, irregularity, line deviation, smoothness, and the extent to which tremor interferes with task performance. However, although being considered the clinical “gold standard” for assessment of tremor, these assessments are inherently subjective; drawing sub-scores in particular may show limited inter-rater reliability, and overall ratings can vary by examiner (9).

Accordingly, multiple approaches have attempted to quantify tremor objectively from writing and drawing (1, 2, 10). Recently, Aharonson et al. (11) presented a rapid, explainable image-processing approach designed for repeated use around FUS treatments. The method applied Sobel edge detection (12) to estimate local gradients, converted these to edge angles, and then computed a “relative orientation angle”. An intentional choice of a single interpretable feature was made for this preliminary stage, and it was noted that future work to compare automated outputs with manual tremor ratings was needed. In the subsequent study, a more elaborate objective tool was introduced, combining image processing and a distance-based scoring framework to quantify tremor change longitudinally after FUS thalamotomy (7). An “optimal solution” reference line between the spiral's guide boundaries was generated. Both the patient trace and the reference line were then sampled to equal point counts, and a performance score was computed as the Manhattan (L1) distance between corresponding sampled points. Using this score, the authors reported significant improvement after FUS in the treated hand and also detected changes in the non-treated hand, alongside evidence suggestive of waning efficacy over time.

Despite the promise of modern computer-vision-based approaches, an important question remains unresolved: how do these automated metrics compare to the clinical “gold standard” clinician-rated spiral drawing scores (8) when both are evaluated on the same observations?

The primary objective of this study was to determine which automated spiral-drawing metric best aligned with clinician-rated tremor severity in patients with essential tremor undergoing unilateral MRgFUS thalamotomy. The primary endpoint was the prediction of the clinician-rated CRST spiral drawing score, recorded on an ordinal 0–4 scale, by the Sobel-based orientation irregularity score, the optimal-solution deviation score, and an additional “common sense” method based on total drawn line length. Secondary endpoints were the ability of these metrics and the overall model to capture the expected treatment-related pattern, defined as a differential pre- to post-MRgFUS change between the treated and non-treated hands. As a supportive internal consistency analysis, we also examined the association between clinician-rated scores for spiral A and spiral B and performed a *post-hoc* reliability sub-sample analysis.

## 2 Methods and Materials

### 2.1 Patients

In total, 132 consecutive patients with ET were initially included in the study, with 106 people included in the final analysis due to incomplete or poor-quality spiral scans. The final cohort included 72 (67.9%) men. Mean patient age was 71.9 ± 7.3 (SD) years, with a range of 43–87 years. Hand dominance was right-sided in 93 patients (87.7%). Among right-hand dominant patients, the dominant side was treated in 91 of 93 patients (97.8%). Among left-hand dominant patients, the dominant side was treated in 12 of 13 patients (92.3%). All patients had medication-refractory tremor and underwent unilateral MRgFUS thalamotomy at Rambam Health Care campus, Haifa, Israel. Patients with baseline drawings for both hands and an immediate 1-week or 1-month follow up (364 and 46 observations, respectively) were included. Additional screening criteria for non-inclusion included conditions which could affect performance, such as reduced visual acuity, upper limb musculoskeletal conditions, cognitive impairment, or uncontrolled hyperthyroidism.

### 2.2 MRgFUS treatments

MRgFUS thalamotomy was performed in a 3-T MRI system (GE Medical Systems, Milwaukee, WI, USA) using an ExAblate Neuro 4,000 device with a silicone membrane (InSightec, Tirat Hacarmel, Israel). The patient's head was immobilized with an MRI-compatible stereotactic frame (Integra Radionics, Burlington, MA, USA). Target temperature was monitored using MR thermometry. Treatment proceeded stepwise: the initial target was planned using a brain atlas and the patient's MRI, followed by low-energy sonication to confirm clinical effect and absence of side effects while gradually increasing target temperature to 45–50 °C. The team reassessed the awake patient repeatedly as the temperature was raised, typically observing resolution of contralateral tremor. Once the target was confirmed without adverse effects, energy was increased to reach 54–60 °C (10–39 s sonications) to create a permanent lesion. If tremor reduction was insufficient or side effects appeared at low temperatures, the target was adjusted until benefit was achieved without unwanted effects.

### 2.3 Ethics

The study followed the Helsinki Declaration (1964) and subsequent amendments and was approved by the Rambam Health Care Campus IRB (4040–17). Data were collected retrospectively and pseudonymized; therefore, written informed consent was not required. The IRB approved the publication, and all authors reviewed and approved the final manuscript.

### 2.4 Spiral drawings dataset

According to CRST, patients received a paper template with two Archimedean spirals. The spirals were labeled A and B, with B being narrower than A. Patients were asked to trace the spirals between the guidelines. They were given guidance not to support their arm while drawing. All the patients were seated at a desk with proper lighting while completing the task. No guidance was given for drawing speed or pressure application on paper. Patients completed the spiral-drawing test before treatment and at multiple follow-up visits, using both the right and left hands each time.

For each template, staff recorded the hand used for drawing and the patient's dominant hand. Templates were scanned at 300 DPI and saved under an anonymized patient/session code. A companion spreadsheet recorded the drawing date, treated hand, and treatment date. Scans then underwent computerized preprocessing to isolate the spirals and remove human markings (patient/clinician notes) and scanning artifacts (e.g., rotation, pixelation). Spiral A and B regions were cropped consistently, resized to 500 × 500 pixels, and converted to grayscale. Spiral A was selected *a priori* as the primary image-analysis target because it is the wider of the two spirals and therefore provides a more robust substrate for trace isolation, skeletonization, and template-based comparison. Although Spiral B is narrower and may be more sensitive to subtle tremor, its narrower spacing also increases susceptibility to boundary effects, overlap with template guide lines, and small differences in cropping, registration, or binarization. To preserve a simple and reproducible primary analysis with minimal additional parameter choices, we therefore used a single predefined spiral rather than combining A and B or selecting between them *post hoc*. Spiral B was retained for an internal consistency analysis of the clinician-rated scores, providing an independent check that ratings of the two spiral tasks reflected a shared clinical construct.

### 2.5 Clinician-rated spiral drawing score

The clinical reference (“gold standard”) was the clinician-rated spiral drawing severity score from the Clinical Rating Scale for Tremor (CRST; Fahn–Tolosa–Marin scale), recorded on the standard ordinal scale ranging from 0 to 4 (0 = no tremor; 4 = severe tremor affecting task performance). This ordinal CRST spiral score served as the dependent variable in all primary models. It was rated by a single highly experienced rater and reflected well the clinical impression documented within each patient's file. The rater is a PhD-certified neurophysiologist with 18 years of experience evaluating Parkinson's disease and essential tremor patients academically and during DBS and MRgFUS procedures. They have authored numerous publications for functional neurosurgery literature and frequently serve as a study rater. The second rater, who served for the *post hoc* reliability sub-sample, is a movement disorders specialist with 7 years of experience and was blinded to treatment.

### 2.6 Image metric computation

All image-derived metrics were computed from the same preprocessed Spiral A images. Following scan-level quality control, each spiral image was cropped from the original template, converted to grayscale, resized to 500 × 500 pixels, and processed to isolate the patient-drawn trace from the printed template and background artifacts. Prior to feature extraction, all spiral scans underwent quality control to determine whether the patient-drawn trace could be reliably isolated from the template and from non-drawing artifacts. A spiral was considered low quality and excluded when computerized preprocessing or visual inspection indicated that valid trace extraction was not possible. Principal causes of rejection included incomplete or missing scans; missing essential metadata such as drawing hand, treatment date, or follow-up status; severe cropping of the spiral region; excessive rotation, skew, blur, pixelation, or poor scan contrast; template misalignment that prevented consistent cropping; and patient or clinician markings, notes, or overwritten lines that overlapped the spiral trace and could not be removed without altering the drawing. Drawings were not excluded solely because of severe tremor, line waviness, retracing, or poor task performance, as these features may reflect true clinical severity. They were excluded only when such features, or superimposed artifacts, prevented reliable separation of the patient trace from the template or background. Three scalar metrics were then computed for each observation: the Sobel-based orientation irregularity score, the optimal-solution deviation score, and total drawn line length. These metrics were computed independently of the clinician-rated CRST spiral score. The Sobel-based orientation irregularity score was computed according to the method described by Aharonson et al. (11). Briefly, Sobel edge detection was applied to the isolated spiral image to estimate the local image gradient at each edge pixel. Horizontal and vertical gradients were obtained by convolving the image (I) with Sobel kernels (*S*_*x*_) and (*S*_*y*_). For each edge pixel with coordinates ((x, y)), the local edge orientation was compared with the radial azimuth from the estimated spiral center ((*C*_*x*_, *C*_*y*_)). The relative orientation angle was therefore defined as follows:


$$\begin{array}{l} \theta \left(x,y\right)=\arctan\left(\frac{S_{x}^{*}I}{S_{y}^{*}I}\right)-arctan\left(\frac{{x-C}_{x}}{{y-C}_{y}}\right). \end{array}$$

where ^*^ denotes the convolution operator. The distribution of θ values across all eligible edge pixels in the spiral trace was summarized by its standard deviation. This value was used as the Sobel-based orientation irregularity score, with larger values indicating a wider dispersion of local edge orientations relative to the expected spiral geometry, and therefore greater drawing irregularity.

The optimal-solution deviation score was computed using the template-based reference-trajectory approach described by Aharonson et al. In brief, the spiral image was binarized, and the patient-drawn trace was isolated after removal of the empty template representation. An ideal reference trajectory was generated from the printed spiral guide boundaries by dividing the template boundary pixels into equal-length segments and connecting equidistant midpoints, thereby creating an “optimal solution” midline between the spiral guide boundaries. The patient trace and the optimal reference line were then resampled to the same number of points. The deviation score was calculated as the aggregate Manhattan distance between corresponding sampled points on the patient trace and the optimal reference trajectory:


$$\begin{array}{l} D=\overset{n}{\underset{i=1}{\sum }}\left(\left|x_{i}-\hat{x_{i}}|+|y_{i}-\hat{y_{i}}\right|\right) \end{array}$$

where (*x*_*i*_, *y*_*i*_) denotes a sampled point on the patient-drawn trace and $\left({\hat{x}}_{i},{\hat{y}}_{i}\right)$ denotes the corresponding point on the optimal reference trajectory. Higher values indicate greater deviation from the expected spiral path.

In addition, we computed the total line length of the patient's spiral trace. Namely, we followed a two-step computational process. First, from the cleaned binary trace mask, we computed a 1-pixel skeleton of the trace (morphological thinning) to minimize sensitivity to pen thickness and scanning artifacts. Second, we estimated length by summing local step distances along the skeleton using 8-connectivity (orthogonal steps counted as 1 pixel; diagonal steps counted as √2 pixels), then converted pixels to physical units using the known scan resolution (300 DPI). The resulting scalar value represents the total inked path length of the spiral trace, where a higher length is interpreted as more oscillatory stroke production and/or retracing, consistent with worse tremor.

### 2.7 Statistical analysis

We analyzed the clinician-rated spiral drawing score (ordinal 0–4) as the primary outcome. Due to the fact that multiple observations were available per subject (up to four) and observations were obtained for both hands across pre- and post-treatment visits, we used an ordered logistic mixed-effects regression (13) to account for within-subject correlation by including a subject-specific random intercept. Fixed effects included the Sobel edge-detection score, the optimal-solution deviation score, and total line length (all continuous predictors), as well as time (post-MRgFUS vs pre-MRgFUS), hand (treated vs non-treated), and their interaction (time × hand), to test whether pre/post change differed between treated and non-treated hands. Overall model significance was evaluated using a Wald chi-square test (14) for the fixed effects, and individual predictors were assessed using Wald tests, reporting effects on the odds-ratio scale, where odds ratios greater than 1 indicate higher odds of a worse/higher clinician score. Because the interaction was prespecified and clinically central, we decomposed it using planned simple comparisons: non-treated versus treated hand at baseline, non-treated versus treated hand post-treatment, and post- versus pre-treatment changes within each hand; these were reported as odds ratios with 95% confidence intervals and *p*-values. Moreover, we derived model-based expected scores by computing predicted probabilities across score categories for each time-by-hand condition and summarizing them into an expected ordinal score. As a sensitivity check, we refit an ordered logistic regression without random effects but with cluster-robust standard errors by subject; conclusions for the key effects were qualitatively consistent with the mixed-effects model. Due to the imbalance in the number of patients who had a 1-week or a 1-month follow-up appointment (364 and 46 observations, respectively), an additional sensitivity analysis was performed by excluding 1-month follow-up observations. To address inter-rater reliability, a *post-hoc* reliability analysis was performed in a subset of 100 randomly chosen spiral-drawing observations. The original CRST spiral A ratings were compared with ratings provided by a second rater. Inter-rater agreement was assessed using quadratic weighted Cohen's kappa.

## 3 Results

### 3.1 Sample and outcome distribution

Observations with missing outcome, time, or treatment status were excluded from the analysis (18 observations). The analyzed data comprised 410 observations taken from 106 subjects with ET (3.9 mean observations per subject; range 1–4). Spiral drawing clinician impression ranged from 0 to 4. The observed distribution was: 0 (22.2%), 1 (29.3%), 2 (27.8%), 3 (14.2%), and 4 (6.6%).

### 3.2 Primary analysis: ordinal mixed-effects model

A mixed-effects ordered logistic regression with a random intercept for subjects was performed to account for repeated measurements for the prediction of clinician-rated spiral drawing scores. Fixed effects included Sobel edge detection score, optimal solution deviation score, line length, time (post-MRgFUS vs pre-MRgFUS), treated-hand (non-treated vs treated), and their time × treated-hand interaction. This model showed overall statistical significance (Wald χ^2^ = 165.15, *p* < 0.001). Among the image-derived predictors, only total line length was independently associated with higher clinician-rated spiral severity. Each unit increase in line length was associated with higher odds of a worse CRST spiral score (OR_(per 1 mm)_ = 1.017, 95% CI 1.007–1.027, z = 3.32, *p* = 0.001). In contrast, the Sobel-based orientation irregularity score (OR = 1.003, 95% CI 0.993–1.013, z = 0.64, *p* = 0.524) and the optimal-solution deviation score (OR = 0.999, 95% CI 0.996–1.002, z = −0.56, *p* = 0.578) were not independently associated with clinician-rated tremor severity.

**Figure 1:**
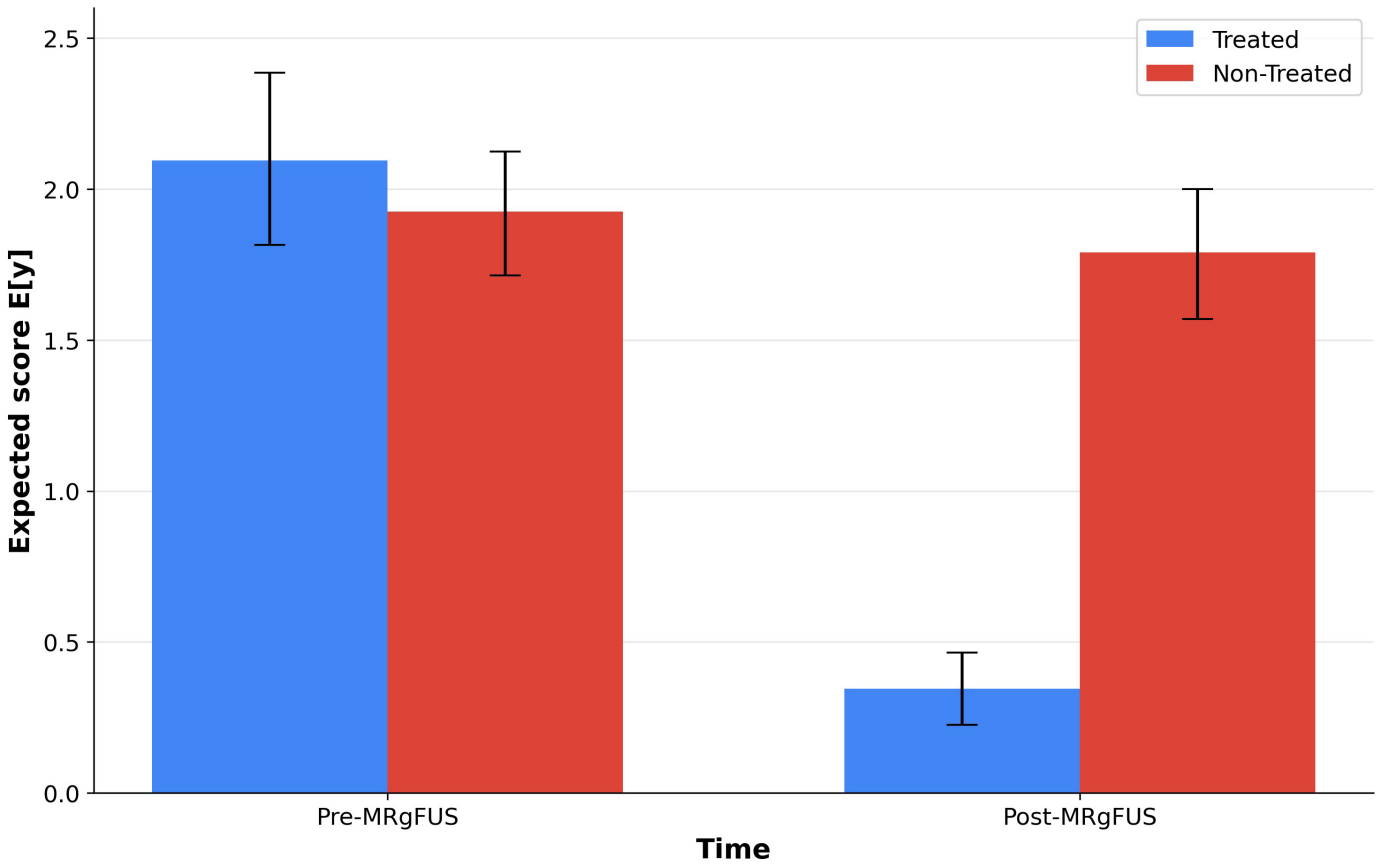
Model-based expected score by time and treated hand. Bars show the expected score, E[y], from the ordinal mixed model, and error bars show 95% confidence intervals. Lower values indicate less severe tremor. The treated and non-treated hands were similar before treatment. After MRgFUS, the treated hand showed a marked reduction in expected tremor score (Post vs Pre within Treated: ΔE[y] = −1.75, 95% CI−2.05 −1.46, *p* < 0.001), whereas the non-treated hand showed no clear change over time. Accordingly, at follow-up, the non-treated hand had a substantially higher expected score than the treated hand (Non-Treated vs Treated at Post-MRgFUS: ΔE[y] = 1.45, 95% CI 1.24 to 1.66, *p* < 0.001). Overall, improvement was mainly confined to the treated hand. Please note that the y-axis shows a probability-weighted average across ordinal score categories (0–4) derived from the mixed-effects ordered logit model, rather than a directly observed raw ordinal score.

The time × treated-hand interaction was significant (χ^2^ = 74.81, *p* < 0.001). Prespecified 2 × 2 comparisons and Odds-ratio scale (ordered logit interpretation) were calculated for further interaction analysis, with OR > 1 meaning higher odds of a worse/higher score. Both methods showed significant tremor reduction in the treated hand only, with the between-hand difference being minimal at baseline but becoming significant at Post-MRgFUS (Table 1, Figure 1).

**Table 1:** Simple odds ratio comparisons.

Condition	*Pre–MRgFUS*	*Post–MRgFUS*
Non–Treated vs. Treated	OR 0.66 (95% CI 0.27–1.63), *p* = 0.364	OR 118.55 (95% CI 49.44–284.24), *p* < 0.001
	**Treated**	**Non–Treated**
Post–MRgFUS vs. Pre–MRgFUS	OR 0.0039 (95% CI 0.0013–0.0116), *p* < 0.001	OR 0.70 (95% CI 0.41–1.19), *p* = 0.188

This table presents prespecified 2 × 2 odds-ratio comparisons based on unadjusted data. The ordinal outcome ranges from 0 to 4, with lower scores indicating better performance. OR > 1 indicates higher odds of a worse score. At baseline, Treated and Non-Treated hands did not differ significantly. Post-MRgFUS, the Non-Treated hand showed markedly higher odds of worse scores than the Treated hand. Within-hand comparisons demonstrated significant improvement only in the Treated hand, while the Non-Treated hand remained unchanged. Together, these findings are consistent with a significant Time × Treatment interaction, indicating a treatment-specific effect.

**Figure 2:**
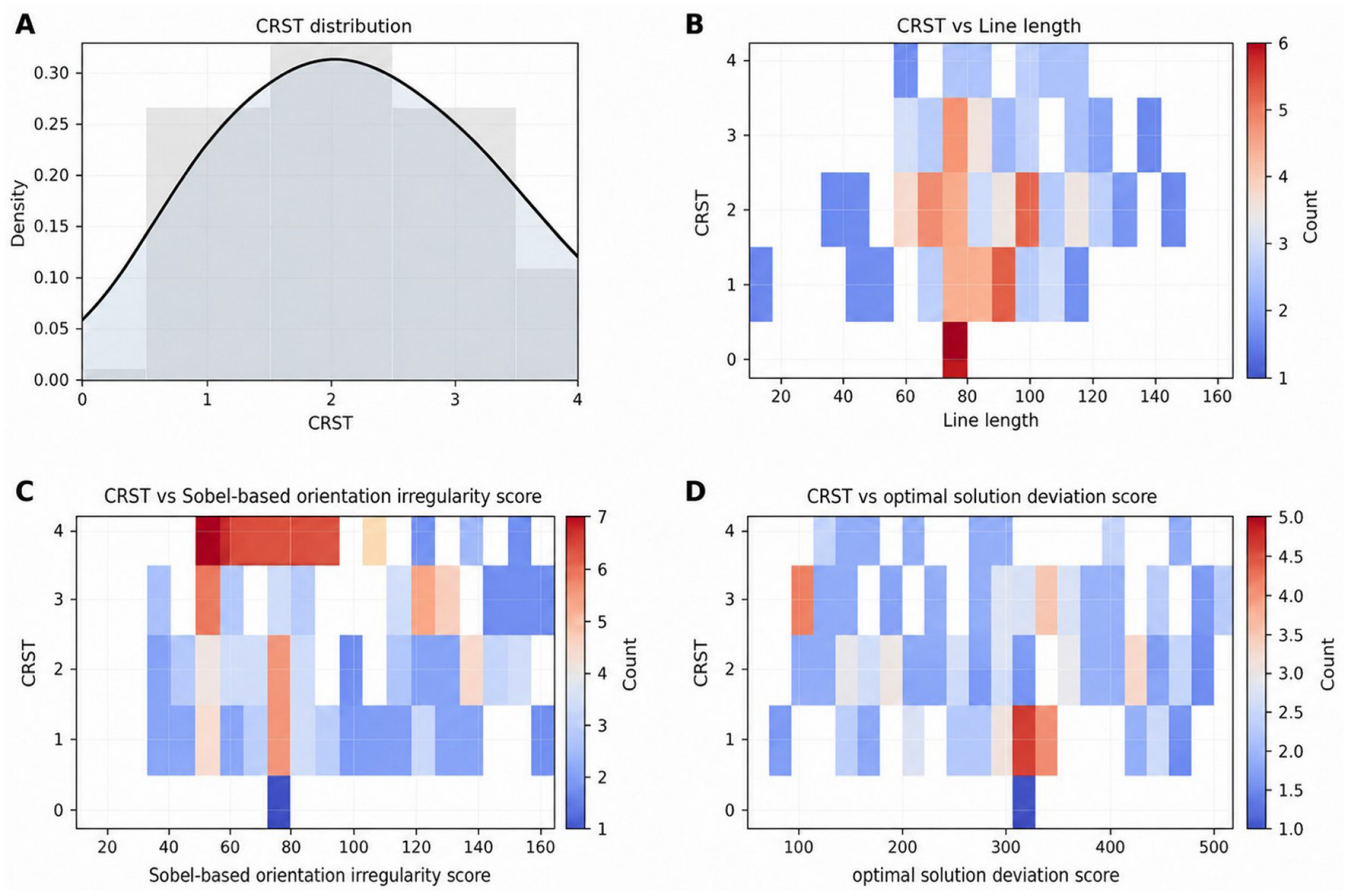
CRST reference distribution and heat maps of image-based spiral metrics vs clinician-rated spiral drawing scores. **(A)** shows the distribution of clinician-rated CRST spiral scores across all observations. Most values are concentrated in the mild-to-moderate range, with fewer observations at the lowest and highest scores. **(B-D)** show heat maps depicting the relationship between CRST score and each quantitative spiral metric. In these panels, color indicates the number of observations within each bin, with warmer colors representing higher counts and cooler colors representing lower counts. **(B)** demonstrates a visible trend between higher CRST scores and greater total line length, suggesting a positive association with clinician-rated tremor severity. In contrast, **(C)** (Sobel-based orientation irregularity score) and **(D)** (optimal solution deviation score) do not show a clear visual trend across CRST categories. Overall, these plots suggest that total line length corresponds more closely with CRST than the two more complex image-derived metrics.

We performed a practical sensitivity check using a non-mixed ordered logistic regression with cluster-robust SEs by subject; results were qualitatively consistent with the mixed model for the key effects. Due to the imbalance in the number of patients who had 1 week or 1 month of follow-up (364 and 46 observations, respectively), an additional sensitivity analysis restricted to the 1-week follow-up cohort yielded results consistent with the primary analysis. Total line length remained independently associated with higher clinician-rated spiral severity [OR = 1.016, *p* = 0.012], whereas the Sobel-based orientation irregularity score [*p* = 0.608] and optimal-solution deviation score [*p* = 0.681] remained non-significant. The time × treated-hand interaction also remained significant [χ^2^ = 68.42, *p* < 0.001], indicating that the expected post-treatment improvement remained confined to the treated hand. Additionally, Figure 2 compares the distribution of the spiral metrics against the clinician-rated spiral drawing scores. The heat maps show a trend in Figure 2B for Line length, but Figures 2C, D do not show a trend for Sobel-based orientation irregularity and optimal solution deviation scores. Figure 2A shows the distribution of the CRST in our cohort for reference.

**Figure 3:**
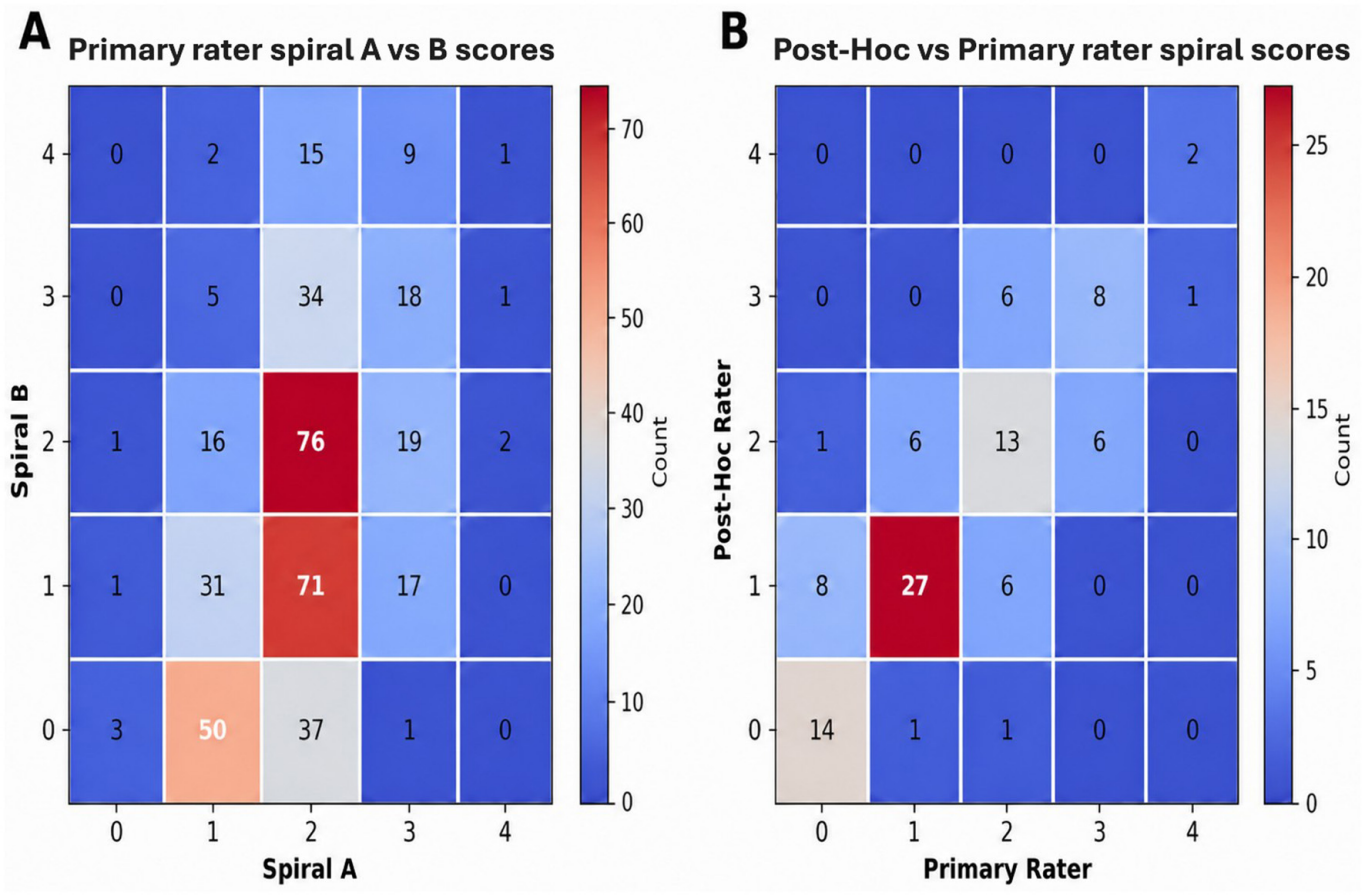
Heat maps for internal consistency and *post-hoc* reliability sub-sample spiral clinical ratings. **(A)** shows a heat map of paired CRST spiral scores assigned by the primary rater to Spiral A and Spiral B. Each cell represents the number of observations with the corresponding combination of Spiral A and Spiral B scores; warmer colors indicate higher counts, and cooler colors indicate lower counts. The concentration of observations along, or near, the diagonal suggests good internal consistency between the two spiral attempts, although perfect agreement is not expected because Spiral A and Spiral B represent separate drawings and may reflect true within-patient performance variability. **(B)** shows a *post-hoc* sub-sample analysis comparing CRST spiral scores assigned by the primary rater and an independent *post-hoc* rater. Numeric values within cells indicate raw counts, with warmer colors indicating higher counts and cooler colors indicating lower counts. The clustering of observations along, or close to, the diagonal suggests good inter-rater reliability.

For an internal consistency check for the clinical ratings, we used the Spearman test to examine the correlation between the clinician-rated spiral drawing scores for spiral A and spiral B, which were highly correlated and significant (r_s_ = 0.88, *p* < 0.001), showing good internal consistency. In the *post-hoc* inter-rater reliability analysis of 100 spiral-drawing observations, quadratic weighted agreement between the two raters was high. The observed weighted agreement was 97.38%, compared with an expected agreement of 86.55%, yielding a high quadratic weighted Cohen's kappa (κ = 0.805, *p* < 0.001). See Figure 3 for visualization of internal consistency (Figure 3A) and *Post-Hoc* reliability (Figure 3B).

## 4 Discussion

In this study, we evaluated whether commonly used, explainable image-based spiral metrics align with the clinical “gold standard” clinician-rated spiral drawing scores and whether they capture the expected pre/post treatment dynamics in the treated hand. We found that among the automated predictors evaluated, only total drawn line length was independently associated with worse clinician-rated spiral severity. In contrast, the Sobel-based orientation irregularity score and the optimal-solution deviation score were not independently associated with the clinician spiral score when modeled concurrently with key clinical factors and repeated-measures structure. At the same time, the model strongly detected the hallmark treatment pattern through a significant time-by-treated-hand interaction: substantial improvement in the treated hand after MRgFUS, without significant changes in the non-treated hand and a pronounced between-hand difference post-treatment. This pattern serves as a validation for the clinician-rated spiral drawing scores along with the expectedly high internal consistency between spiral A and spiral B ratings and the strong agreement in our *post-hoc* sub-sample analysis.

An additional consideration concerns prior reports of apparent improvement in the non-treated hand after unilateral MRgFUS (7). Although such findings may suggest bilateral tremor benefit, they should be interpreted cautiously in light of the present results. One possible explanation is that different image-derived metrics may vary in their sensitivity to tremor-related features and may also capture non-tremor factors related to drawing quality, task execution, or image preprocessing. In addition, apparent contralateral improvement may not necessarily reflect a direct therapeutic effect on the untreated limb, but rather an indirect downstream effect of reducing tremor in the more severely affected or functionally dominant hand. In clinical practice, some patients describe the less affected limb as trembling secondarily when it is mechanically or behaviorally coupled to the more tremulous hand, for example during bimanual tasks or when the dominant hand “drags” the opposite limb into oscillatory movement. Once the primary tremor is reduced and the more affected hand stabilizes, this transmitted or coupled tremor may diminish, creating the appearance of contralateral benefit. In the present analysis, the absence of a significant pre- to post-MRgFUS change in the non-treated hand supports a more conservative interpretation: the dominant treatment signature was improvement in the treated hand, whereas any apparent past contralateral effects require cautious interpretation.

These findings have two main implications. First, not all plausible computer-vision spiral features behave as surrogates for clinician impression when evaluated head-to-head on the same observations. The lack of independent association for the Sobel and optimal-solution deviation scores suggests that, as currently operationalized, these metrics may be more sensitive to aspects of drawing quality, scanning artifacts, or template-processing assumptions than to the clinical construct captured by the CRST spiral rating. Alternatively, they may capture tremor characteristics (e.g., high-frequency micro-oscillations, edge-angle dispersion) that are not emphasized by clinicians when assigning CRST scores, which integrate global appearance, smoothness, and functional legibility. Second, the strong performance of a simple line-length feature supports the idea that clinically salient tremor severity in spiral drawings may be dominated by gross path inefficiency, such as extra oscillatory excursion, retracing, and exaggerated curvature, which length aggregates naturally. In this sense, the “common sense” metric may act as a robust proxy for the visual magnitude of deviation that clinicians intuitively rate, while remaining relatively resilient to some preprocessing idiosyncrasies. This interpretation should be considered in light of an important limitation of the validation target. As the CRST itself (and more specifically, the drawing sub-scores) serves as a subjective measurement of tremor with imperfect reliability (9), it remains a widely used clinical reference standard for tremor severity and is currently the most practical comparator available in retrospective scanned-spiral datasets. Therefore, stronger agreement with CRST should be interpreted as better alignment with clinician-rated tremor severity, rather than as definitive proof that one metric captures the full construct of tremor more accurately than another.

This study is not without limitations. First, the retrospective design and reliance on scanned paper spirals introduce variability from scanning resolution, pen type, and template alignment, all of which may differentially affect image-derived scores. Second, we focused on association with an ordinal clinician impression rather than predicting continuous kinematic tremor features that tablet-based approaches can capture. We could not account for drawing speed, pauses, hesitation, or pressure. Therefore, our results should be interpreted as a practical marker of tremor severity rather than the only measurement needed for assessment. Third, the reliance on a single rater is prone to bias, which we addressed by performing both internal consistency and *post-hoc* reliability analyses.

Spiral-based assessment may have practical value across the MRgFUS treatment pathway. Pre-operatively, objective spiral metrics can provide a standardized baseline that complements clinical examination and helps document tremor severity. During treatment, a rapid quantitative measure could support the treating physician during sequential sonications by adding an objective estimate of tremor reduction to bedside clinical judgment. This may help determine whether sufficient benefit has been achieved or whether further sonication is warranted. During follow-up, objective spiral quantification may make pre- to post-treatment differences clearer and more reproducible and may help monitor partial response, waning benefit, or tremor recurrence. Thus, spiral-based metrics should not replace clinical evaluation, but may improve the objectivity and consistency of tremor assessment before, during, and after MRgFUS.

## 5 Conclusions

Taken together, these results show that objective spiral features can track clinically relevant tremor change after MRgFUS, with the model capturing the expected improvement specifically in the treated hand. Among the tested automated measures, simple line length aligned best with clinician-rated severity, suggesting that low-complexity, interpretable metrics may be the most practical starting point for routine pre/post quantification and future tool development. Future work should evaluate reliability across raters and centers, incorporate standardized scanning controls, and consider hybrid models that combine robust geometric features (such as length) with targeted frequency-sensitive features such as tablets or wearables, potentially improving sensitivity to subtle changes while preserving alignment with clinically meaningful severity. Validation against multiple scales such as Bain Findley spiral scores (BFS) and The Essential Tremor Rating Assessment Scale (TETRAS) and patient-reported measures would further clarify which automated metrics best reflect real-world benefit after MRgFUS.

## Data Availability

The raw data supporting the conclusions of this article will be made available by the authors, without undue reservation.
